# Observations on Red Mark Syndrome in juvenile rainbow trout farmed in RAS system

**DOI:** 10.1111/jfd.13707

**Published:** 2022-08-14

**Authors:** Massimo Orioles, Elena Saccà, Mathjis Metselaar, Michela Bulfoni, Daniela Cesselli, Marco Galeotti

**Affiliations:** ^1^ Department of Agricultural, Food, Environmental and Animal Sciences, Veterinary Pathology Unit University of Udine Udine Italy; ^2^ Aquatic Vets Ltd. Stirling Scotland; ^3^ Department of Medicine University of Udine Udine Italy; ^4^ Department of Medicine University of Udine, Italy Institute of Clinical Pathology, ASUFC Udine Italy

**Keywords:** digital droplet PCR, Midichloria‐like organism, rainbow trout, recirculating aquaculture system, red mark syndrome

Red mark syndrome is a non‐lethal skin disease, reported to affect farmed rainbow trout over 50 g worldwide (Metselaar et al., 2022). As reported by the results of the 25th Annual Workshop of the European Reference Laboratories for Fish Diseases (DTU‐AQUA, Denmark), RMS remains to date a major concern in rainbow trout farming. This syndrome has been studied extensively both in field and experimental cases, and diagnostic features have been established (Galeotti, Sarli, et al., 2021; Jorgensen et al., 2019; Oidtmann et al., 2013; Verner‐Jeffreys et al., 2008). Slovenia and Bosnia‐Herzegovina were the most recent European areas where RMS was reported to outbreak in intensive aquaculture farms (Galeotti, Manzano, et al., 2017; Galeotti, Volpatti, et al., 2021). Even though no aetiological agent has been proven through Koch's postulates, RMS has been consistently associated with the presence of Midichloria‐like organisms which have been visualized in situ by transmission electron microscopy both in field and experimental cases (Cafiso et al., 2016; Galeotti, Ronza, & Beraldo, 2017; Metselaar et al., 2020; Orioles et al., 2022b).

The outbreak under study occurred in a Slovenian rainbow trout recirculating aquaculture system (RAS) in March 2022 (water temperature 11–12°C). Eggs came from a local domestic supplier. Fish were reared in concrete basins from 10 up to 150 g before being transferred to another farm to reach commercial size. Rainbow trout were fed a standard commercial‐pelleted diet.

Water was taken from a local spring, and no other fish farms were supplied from the same water source either upstream or downstream.

About 50% of fish were showing signs compatible with RMS as shown in Figure 1. No mortality was observed, and affected fish had normal food intake and growth rate.

**FIGURE 1:**
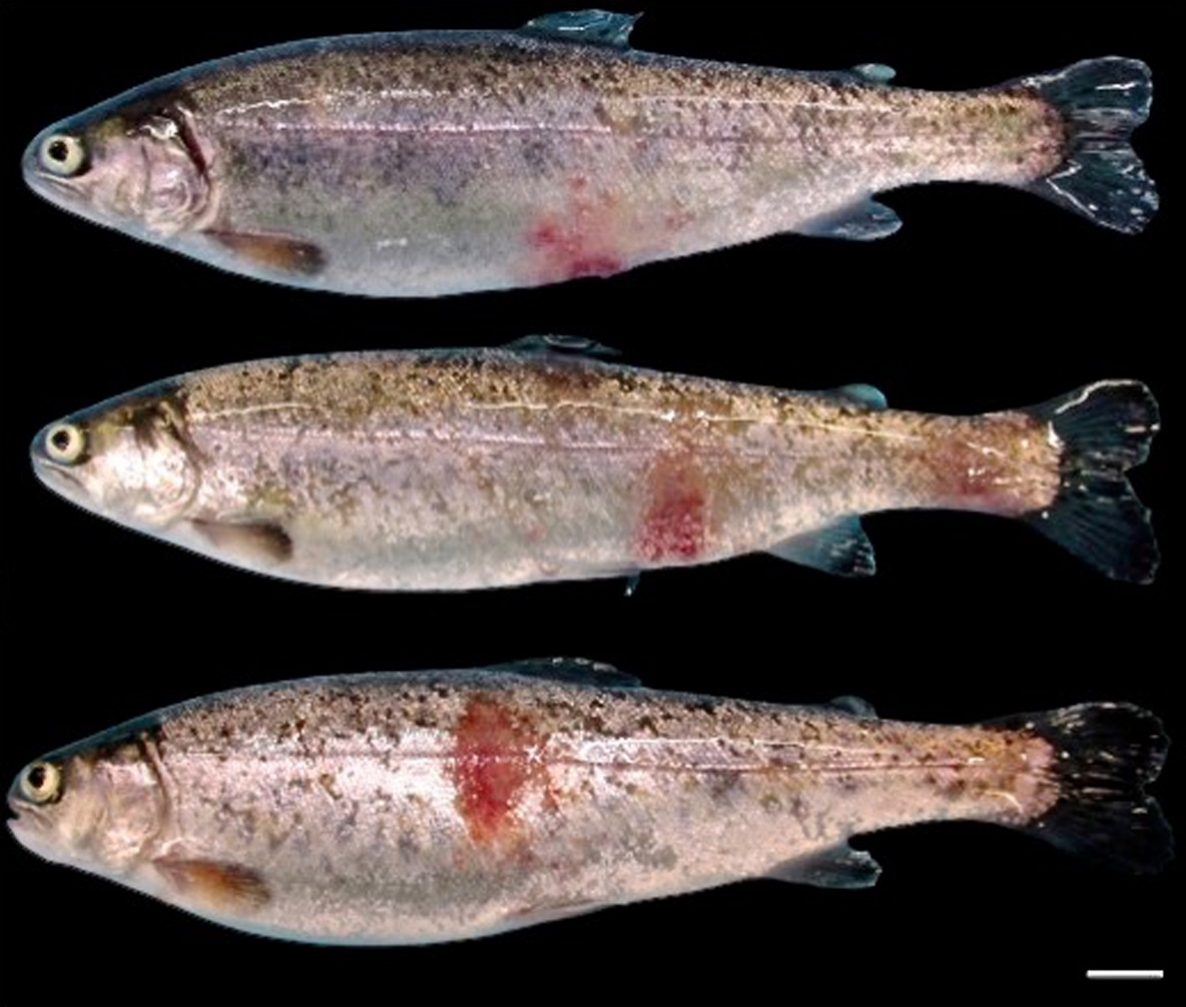
Typical RMS macroscopic skin lesions. Gross skin lesions ranging from 1 to 2 cm were round to oval, reddish and covered by serous exudate associated with multifocal haemorrhages lesions were focal (8/10) to multifocal (2/10) placed on ventral flanks and abdomen. Desquamation and skin ulcers were present in 3 out of the 10 trout. Bar = 1 cm

Ten symptomatic fish were sampled and killed with an overdose of MS222 for investigation purposes. Fish's weight ranged from 35 to 90 g and they were all in good body condition. Approximately, 1 ml of blood was collected from the caudal vein and put in a 1.5 ml sterile plain tube where it was allowed to clot to extract DNA. Samples of internal organs (gills, intestine, spleen, liver, kidney and heart) and samples from skin lesions and spleen were taken for histological and molecular biology analysis, respectively. Tissues obtained for histological examination from skin/muscle and internal organs were fixed in buffered formaldehyde and then processed by an automatic histoprocessor (TISBE, Diapath, Italy) to be embedded in paraffin (ParaplastPlus, Diapath). Serial 5 μm sections were stained with haematoxylin–eosin.

QIAmp DNA Mini extraction Kit (Qiagen, Hilden, Germany) was used on skin and spleen samples and on blood clots, using Tissue Lyser II equipment (Qiagen, Hilden, Germany). The concentration and purity of the extracted DNA were assessed using a ‘NanoDrop One’ spectrophotometer (Thermo Scientific, Waltham, MA, USA), and the DNA was then sent to the digital droplet PCR (ddPCR) analysis.

The ddPCR method was performed on the DNA extracted in accordance with recent literature (Orioles, Bulfoni, et al., 2022) and manufacturer's instructions (Bio‐Rad, USA).

Gross skin lesions ranging from 1 to 2 cm were round to oval, reddish and covered by serous exudate associated with multifocal haemorrhages lesions were focal (8/10) to multifocal (2/10) placed on ventral flanks and abdomen. Desquamation and skin ulcers were present in 3 out of the 10 trout.

Necroscopy revealed splenomegaly in two fish. No abnormalities were detectable in the other organs. Cytology of skin and gills did not reveal the presence of parasites.

Histological examination of skin samples revealed a marked lymphocytic and macrophagic inflammatory infiltration involving all skin layers, including the hypodermis and muscular tissue (Figure 2); the infiltration of lymphocytes and macrophages in the stratum spongiosum of the derma was moderate to severe with frequent scale pockets oedema and cellular infiltration; frequent scale reabsorption and thickening of the stratum compactum of the derma, infiltrated by lymphocytes and macrophages was observed in most severe cases (Figure 2). The underlying muscular tissue was also affected by lymphocytic and macrophagic cellular infiltration in moderate and severe cases. The two macroscopically abnormal spleens showed severe sinusoid congestion. No evidence of bacteria was observed. Histological examination of remaining organs did not reveal significant abnormalities.

**FIGURE 2:**
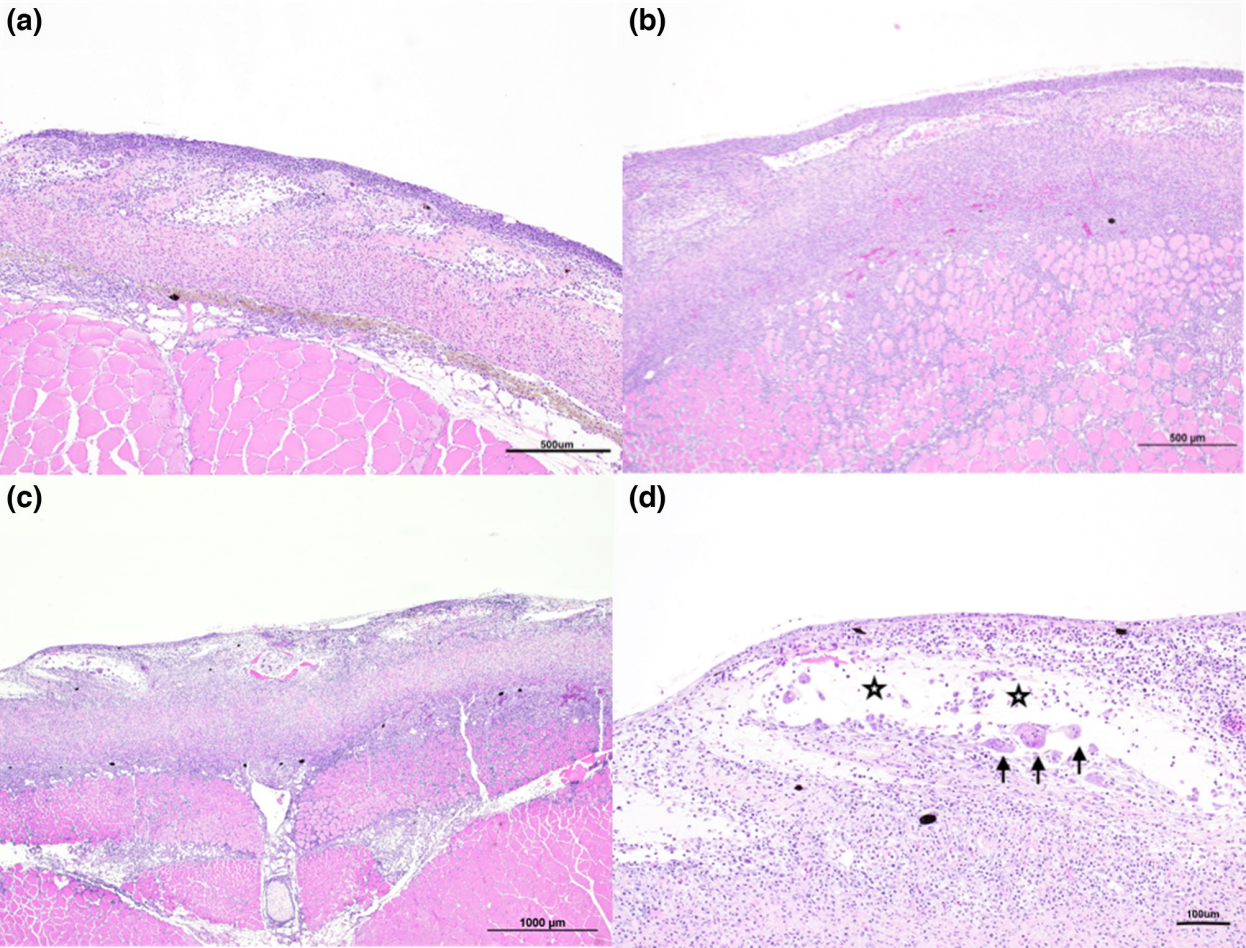
(a–d): H&E sections. Histological features of RMS skin lesion. In early moderate cases (a), infiltration of the lymphocytic and macrophagic inflammatory cells involve predominantly dermis spongiosum and hypoderma; the same type of inflammatory infiltration involves all skin layers with progressive infiltration and thickening of dermis compactum, hypodermis and muscles in moderate and severe cases (b and c). Scale pocket (black stars) oedema, inflammatory infiltration and scale reabsorption by giant multinucleated cells (black arrows) are frequently observed (d)

Skin lesions were classified as moderate or severe as reported recently in the literature (Orioles, Galeotti, et al., 2022). Results of the molecular biology analysis are reported in Table 1.

**TABLE 1:** Digital droplet PCR detection of MLO DNA from skin, spleen, blood and water samples

Case n°	MLO DNA copies in skin (ddPCR)	MLO DNA copies in spleen (ddPCR)	MLO DNA copies in blood (ddPCR)
1	250	7.5	negative
2	4300	2900	10
3	6560	4200	22
4	1560	324	negative
5	92	628	3
6	1700	410	negative
7	344	74	5
8	9500	4340	38
9	1000	334	4
10	54	16	negative

Considering reported criteria for skin disorders in rainbow trout (Oidtmann et al., 2013) and the findings reported in this study, the skin lesions were consistent with RMS. This is the first report of RMS affecting rainbow trout in a RAS system and the evidence that RMS could affect rainbow trout under 50 g (smallest was 35 g) of weight, developing severe lesions similar to what occurs in market size fish. The manuscript by Oh et al. (2019) describes several cases of the disease in rainbow trout in South Korea, in which macroscopic lesions resembling RMS can be seen in juvenile rainbow trout weighed less than 30 g. The high level of mortality observed by Oh et al. (2019) does not reflect the classic case definition for RMS though and further investigations were suggested as necessary to confirm the RMS status of these fish (Metselaar et al., 2022).

Even though final confirmation would require further analysis, in this case, eggs and lack of biosecurity measures might have been the possible sources of infection. The results confirm ddPCR as a very sensitive method to assess MLO DNA quantitatively; blood may be an appropriate matrix where to detect MLO and might be used for culturing this organism as MLO may have a bacteriemic phase, in its strictest sense, and distributes in different organs. The pathogenesis of RMS and the potential role of MLO have yet to be elucidated, and no conclusions can be drawn from the present study.

## CONFLICT OF INTEREST

The authors declare that they have no known competing financial interests or personal relationships that could have appeared to influence the work reported in this paper.

## Data Availability

The data that support the findings of this study are available from the corresponding author upon reasonable request." cd_value_code="text
